# Clinical implications of serum total VEGF-A and VEGF-A isoforms in patients with *EGFR*-mutated advanced non-small cell lung cancer treated with EGFR-TKIs

**DOI:** 10.1007/s10147-026-03083-2

**Published:** 2026-06-09

**Authors:** Tetsu Hirakawa, Kakuhiro Yamaguchi, Kiyofumi Shimoji, Shinjiro Sakamoto, Yasushi Horimasu, Takeshi Masuda, Taku Nakashima, Hiroshi Iwamoto, Hironobu Hamada, Noboru Hattori

**Affiliations:** 1 Department of Molecular and Internal Medicine, Graduate School of Biomedical and Health Sciences, Hiroshima University, 1-2-3 Kasumi, Minami-Ku, Hiroshima, 734-8551 Japan; 2 Department of Respiratory Medicine, Hiroshima University Hospital, Hiroshima, Japan; 3 Department of Physical Analysis and Therapeutic Sciences, Graduate School of Biomedical and Health Sciences, Hiroshima University, Hiroshima, Japan

**Keywords:** Epidermal growth factor receptor, Tyrosine kinase inhibitor, Vascular endothelial growth factor-A, VEGF_121_, Non-small cell lung cancer, Biomarker

## Abstract

**Background:**

Vascular endothelial growth factor (VEGF)-A, which is overexpressed in epidermal growth factor receptor (*EGFR*)-mutated non-small cell lung cancer (NSCLC), has been implicated in resistance to EGFR-tyrosine kinase inhibitors (TKIs). VEGF-A comprises several isoforms, including VEGF_121_ and VEGF_165_. However, the clinical potential as blood biomarkers in EGFR-TKI treatment remains unclear.

**Methods:**

We retrospectively analyzed 64 patients with advanced *EGFR*-mutated NSCLC who received EGFR-TKI monotherapy at Hiroshima University Hospital between November 2009 and August 2023. Pre-treatment serum levels of total VEGF-A (tVEGF-A), VEGF_121_, and VEGF_165_ were measured using enzyme-linked immunosorbent assays. The association of these biomarkers with the objective response rate (ORR), progression-free survival (PFS), and overall survival (OS) was analyzed.

**Results:**

The ORR was 78.1%, and the median PFS and OS were 7.5 and 31.8 months, respectively. Serum VEGF_121_ levels were significantly higher in non-responders than in responders (median, 446.6 vs. 322.1 pg/mL, *p* = 0.043), whereas no significant differences were observed for tVEGF-A and VEGF_165_ levels. Receiver operating characteristic analysis identified an optimal serum VEGF_121_ cut-off level of 339.3 pg/mL (AUC: 0.678, sensitivity: 52.0%, specificity: 92.9%). Patients with higher serum VEGF_121_ (> 339.3 pg/mL) had significantly lower ORR (64.9% vs. 96.3%, *p* = 0.003), shorter PFS (median, 6.2 vs. 14.4 months, *p* = 0.026), and shorter OS (median, 27.6 months vs. not reached, *p* = 0.040) than those with lower serum VEGF_121_ (≤ 339.3 pg/mL).

**Conclusion:**

Higher levels of serum VEGF_121_ were associated with lower EGFR-TKI efficacy and poorer prognosis in patients with *EGFR*-mutated NSCLC.

## Introduction

Epidermal growth factor receptor (EGFR)-tyrosine kinase inhibitors (TKIs) have demonstrated efficacy in patients with *EGFR*-mutated non-small cell lung cancer (NSCLC) [1]. EGFR-TKI monotherapy yields a relatively high objective response rate (ORR) of approximately 76%–80% in patients with previously untreated *EGFR*-mutated advanced NSCLC; nevertheless, a subset of patients does not derive therapeutic benefit [2]. Several potential mechanisms of treatment resistance to EGFR-TKIs have been reported. For example, in *EGFR*-mutated NSCLC, vascular endothelial growth factor (VEGF)-A expression has been reported to be upregulated [3]. Preclinical studies indicate that the VEGF and EGFR pathways share common downstream signaling and can also operate independently during oncogenesis [4]. In clinical trials, the addition of VEGF/VEGF receptor (VEGFR) inhibitors to EGFR-TKI improved clinical outcomes [5, 6]. In addition, a previous study demonstrated that serum VEGF-A levels declined after the initiation of osimertinib [7]. Furthermore, post-treatment reduction of serum VEGF levels has been reported to be significantly associated with longer progression-free survival (PFS) of EGFR-TKI treatment in patients with *EGFR*-mutated NSCLC [8]. These observations suggest that pre-treatment circulating VEGF-A may be associated with EGFR-TKI efficacy and prognosis in patients with *EGFR*-mutated NSCLC. However, this relationship has not yet been fully elucidated.

The VEGF-A gene comprises eight exons separated by seven introns [9], and alternative splicing of exons 5–8 generates multiple VEGF-A isoforms, including VEGF_121_, VEGF_165_, and VEGF_189_ [10–12]. Among these, VEGF_121_ and VEGF_165_ are predominantly secreted by tumor cells [13]. In vivo, VEGF_121_ is more involved in tumor growth than VEGF_165_ [14]. We previously reported that higher levels of serum VEGF_121_ are associated with lower response to immune checkpoint inhibitors (ICIs) in patients with NSCLC [15].

Therefore, this study aimed to elucidate the associations of serum total VEGF-A (tVEGF-A) and its major isoforms, VEGF_121_ and VEGF_165_, with the treatment efficacy and prognosis in patients with *EGFR*-mutated NSCLC treated with EGFR-TKIs.

## Materials and methods

### Study population and design

**Fig. 1:**
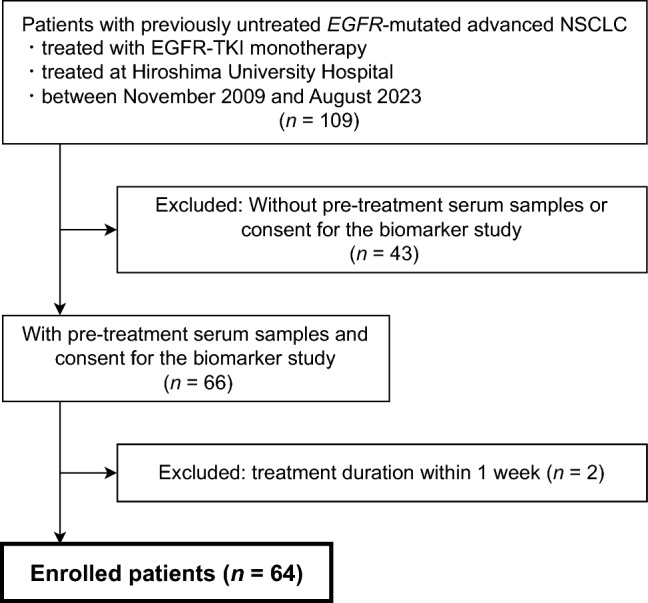
Flowchart of patient enrollment. *Abbreviations*: EGFR, epidermal growth factor receptor; TKI, tyrosine kinase inhibitor; NSCLC, non-small cell lung cancer

This study retrospectively screened 109 patients with previously untreated *EGFR*-mutated advanced NSCLC who were treated with EGFR-TKI monotherapy (gefitinib, erlotinib, afatinib, or osimertinib) at the Department of Respiratory Medicine, Hiroshima University Hospital, between November 2009 and August 2023 (Fig. 1). Of these, 43 patients were excluded due to the unavailability of preserved pre-treatment serum samples or the inability to obtain informed consent for the biomarker analysis. Two patients who discontinued EGFR-TKI treatment within one week of initiation were excluded. Ultimately, 64 patients were analyzed in this study.

This study was conducted in accordance with the principles of the Declaration of Helsinki and was approved by the Ethics Committee of Hiroshima University Hospital (E2004-0326). Written informed consent was obtained from all the participants.

### Evaluations of antitumor treatment response

In this study, the associations of serum tVEGF-A and VEGF-A isoforms with ORR, PFS, and overall survival (OS) were evaluated. Complete response (CR), partial response (PR), stable disease (SD), progressive disease (PD), and not evaluable (NE) were determined based on the Response Evaluation Criteria in Solid Tumors 1.1 [16]. Based on the treatment response, CR and PR were defined as responders, and SD, PD, and NE were defined as non-responders. The ORR was defined as the proportion of patients who achieved CR or PR. PFS was defined as the time from the start of each treatment until PD or death. OS was defined as the time from the start of each treatment until death or the last follow-up. Patients who failed to follow up were censored on the date of their last known survival. The cut-off date for data collection was October 2025.

### Measurement of serum tVEGF-A and VEGF-A isoforms

Serum samples were collected prior to EGFR-TKI administration and stored at − 80 °C. Serum tVEGF-A levels were determined using an enzyme-linked immunosorbent assay (ELISA) system developed by Shino-test Corporation. Polystyrene microtiter plates were coated and incubated with 100 μL of anti-human VEGF-A polyclonal antibody (R&D Biosystems, Minneapolis, MN) in phosphate-buffered saline (PBS) overnight at 4 °C. The plates were washed three times with PBS containing 0.05% Tween 20, and the remaining binding sites in the wells were blocked by incubating the plates for 2 h with 400 μL/well PBS containing 0.5% casein. After the plates were washed, 100 μL of each dilution of the calibrator and samples (1:1 dilution in 0.2 mol/L Tris pH 8.5 and 0.15 mol/L sodium chloride containing 1% casein) were added to the wells. The plates were then incubated for 15 h at 25 °C. The plates were washed again and incubated with 100 μL/well of peroxidase-conjugated anti-human VEGF-A monoclonal antibody (R&D Biosystems, Minneapolis, MN) for 2 h at 25 °C. After another washing step, the chromogenic substrate 3,3’,5,5’-tetra-methylbenzidine (Dojindo Laboratories, Kumamoto, Japan) was added to each well. The reaction was terminated with sulfuric acid, and the absorbance was measured at 450 nm using a microplate reader (Model 680, Bio-Rad, Irvine, CA). VEGF_121_ and VEGF_165_ levels were measured using ELISA kits (Shino-Test, Kanagawa, Japan) [11].

### Statistical analysis

Values are expressed as median (interquartile range [IQR]), unless stated otherwise. Differences among the groups were examined using Pearson’s chi-square and Mann–Whitney U tests. Correlations between variables were ascertained using Spearman correlation coefficients. Receiver operating characteristic (ROC) curve analysis was performed to identify the optimal cut-off levels of serum tVEGF-A and VEGF-A isoforms for predicting the response to EGFR-TKIs. Kaplan–Meier analysis and the log-rank test were used to evaluate 2-year PFS and 3-year OS, and the median PFS and OS intervals with corresponding 95% confidence intervals (CIs) were calculated. Univariate and multivariate logistic regression analyses and Cox proportional hazard models were used to identify independent factors related to ORR, PFS, and OS, respectively. Statistical significance was set at *p* < 0.05. All data analyses were performed using the JMP statistical software version 18.2.1 (SAS Institute Inc., Cary, NC, USA).

## Results

### Patient characteristics

The baseline patient characteristics are summarized in Table 1. Among the 64 patients, the median age was 69 years (59–77), 26 (40.6%) were male, and 58 (90.6%) had an Eastern Cooperative Oncology Group Performance Status (ECOG PS) 0–1. *EGFR* mutations were exon 19 deletion in 31 (48.4%), L858R mutation in 26 (40.6%), and uncommon mutations in 7 (10.9%). At baseline, brain metastasis was observed in 24 (37.5%), pleural effusion in 29 (45.3%), and liver metastasis in 12 (18.8%). The administered EGFR-TKIs were gefitinib in 23 (35.9%), erlotinib in 12 (18.8%), afatinib in 7 (10.9%), and osimertinib in 22 (34.4%).

**Table 1:** Baseline characteristics

	All patients	Responder	Non-responder	*p*-value
Subjects, n	64	50	14	
Age, years	69 (59–77)	69 (61–77)	69 (56–79)	0.871
Sex				0.847
Male, n (%)	26 (40.6)	20 (40.0)	6 (42.9)	
Female, n (%)	38 (59.4)	30 (60.0)	8 (57.1)	
Smoking history				0.847
Current or Former, n (%)	26 (40.6)	20 (40.0)	6 (42.9)	
Never, n (%)	38 (59.4)	30 (60.0)	8 (57.1)	
BMI	22.2 (19.9–24.5)	22.0 (20.0–24.6)	22.7 (19.7–24.1)	0.846
ECOG PS				0.005^**^
0–1, n (%)	58 (90.6)	48 (96.0)	10 (71.4)	
≥ 2, n (%)	6 (9.4)	2 (4.0)	4 (28.6)	
Stage				0.749
III, n (%)	1 (1.6)	1 (2.0)	0 (0.0)	
IV, n (%)	62 (96.9)	48 (96.0)	14 (100.0)	
Recurrence, n (%)	1 (1.6)	1 (2.0)	0 (0.0)	
Histological type				0.594
Adeno, n (%)	63 (98.4)	49 (98.0)	14 (100.0)	
Adenosquamous, n (%)	1 (1.6)	1 (2.0)	0 (0.0)	
*EGFR* mutation				0.241
Exon 19 deletion, n (%)	31 (48.4)	27 (54.0)	4 (28.6)	
L858R mutation, n (%)	26 (40.6)	18 (36.0)	8 (57.1)	
Uncommon mutation^†^, n (%)	7 (10.9)	5 (10.0)	2 (14.3)	
Baseline tumor metastasis				
Brain metastasis, n (%)	24 (37.5)	19 (38.0)	5 (35.7)	0.876
Pleural effusion, n (%)	29 (45.3)	20 (40.0)	9 (64.3)	0.107
Liver metastasis, n (%)	12 (18.8)	9 (18.0)	3 (21.4)	0.771
EGFR-TKI				0.509
Gefitinib, n (%)	23 (35.9)	19 (38.0)	4 (28.6)	
Erlotinib, n (%)	12 (18.8)	9 (18.0)	3 (21.4)	
Afatinib, n (%)	7 (10.9)	4 (8.0)	3 (21.4)	
Osimertinib, n (%)	22 (34.4)	18 (36.0)	4 (28.6)	

Data are presented as median (interquartile range) unless stated otherwise

^†^Uncommon mutation included exon 19 insertion (n = 2), L861Q (n = 2), L861R (n = 1), L858R + T790M (n = 1), G719S + T790M (n = 1)

^**^p < 0.01, comparison between responder and non-responder using the Mann–Whitney *U* test or Pearson’s chi-square test

Adeno, adenocarcinoma; Adenosquamous, adenosquamous carcinoma; BMI, body mass index; ECOG PS, Eastern Cooperative Oncology Group Performance Status; EGFR, epidermal growth factor receptor; TKI, tyrosine kinase inhibitor

The median observation period was 28.3 (10.4–44.3) months. The treatment responses to EGFR-TKI monotherapy were classified as CR in 0 (0.0%), PR in 50 (78.1%), SD in 9 (14.1%), PD in 3 (4.7%), and NE in 2 (3.1%), with an ORR of 78.1%. The median PFS and OS were 7.5 (95% CI: 5.7–11.7) and 31.8 (95% CI: 24.5–42.9) months, respectively. Based on treatment response, responders (CR or PR) comprised 50 patients (78.1%), and non-responders (SD, PD, or NE) comprised 14 patients (21.9%). Baseline patient characteristics according to treatment response are also shown in Table 1. ECOG PS 0–1 was significantly more frequent in responders than in non-responders (96.0% vs. 71.4%, *p* = 0.005). No other variables differed significantly between the two groups.

### Serum levels of tVEGF-A and VEGF-A isoforms stratified by the response to EGFR-TKI

The median serum levels of tVEGF-A, VEGF_121_, and VEGF_165_ were 445.2 (206.9–687.9), 383.5 (205.7–527.9), and 174.2 (110.9–262.3) pg/mL, respectively. These markers were positively correlated with white blood cell (WBC) count, platelet count, and C-reactive protein (CRP) (Supplementary Table 1).

**Fig. 2:**
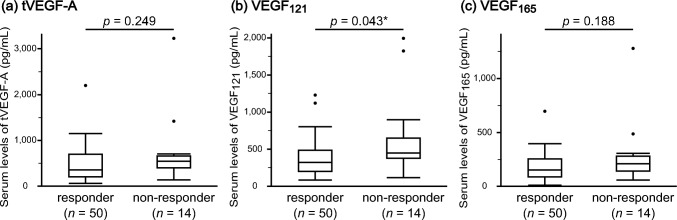
Comparison of serum total vascular endothelial growth factor (tVEGF)-A and VEGF-A isoforms levels between the responder and the non-responder groups. Serum VEGF_121_ levels were significantly higher in the non-responder group than in the responder group (446.6 [377.5–649.2] vs. 322.1 [202.2–487.3] pg/mL, *p* = 0.043). In contrast, no significant differences were observed in serum tVEGF-A (546.5 [400.4–661.2] vs. 354.7 [204.2–695.2] pg/mL, *p* = 0.249) and VEGF_165_ levels (210.8 [141.6–278.9] vs. 150.6 [87.4–255] pg/mL, *p* = 0.188) between the two groups. Boxes represent the interquartile range (25th–75th percentiles), solid lines within the boxes indicate the medians, and whiskers denote the 10th and 90th percentiles. Outliers are plotted as individual points. ^*^p < 0.05, Mann–Whitney U test. *Abbreviations*: tVEGF, total vascular endothelial growth factor; VEGF, vascular endothelial growth factor

Serum VEGF_121_ levels were significantly higher in non-responders than in responders (446.6 [377.5–649.2] vs. 322.1 [202.2–487.3] pg/mL, *p* = 0.043) (Fig. 2b). However, no significant differences were observed in serum tVEGF-A (546.5 [400.4–661.2] vs. 354.7 [204.2–695.2] pg/mL, *p* = 0.249) and VEGF_165_ levels (210.8 [141.6–278.9] vs. 150.6 [87.4–255.0] pg/mL, *p* = 0.188) between the two groups (Fig. 2a, c).

### Stratification of ORR and PFS by serum levels of tVEGF-A and VEGF-A isoforms

ROC curve analysis revealed that the areas under the curve (AUCs) of serum tVEGF-A, VEGF_121_, and VEGF_165_ for predicting response to EGFR-TKI were 0.601 (95% CI: 0.434–0.748), 0.678 (95% CI: 0.509–0.810), and 0.616 (95% CI: 0.449–0.759), respectively (**Supplementary Fig. 1**). The cut-off levels of serum tVEGF-A and VEGF-A isoforms were 392.8, 339.3, and 120.3 pg/mL, respectively. Among them, the highest AUC was observed for VEGF_121_, for which the optimal cut-off level of 339.3 pg/mL provided 52.0% sensitivity and 92.9% specificity.

**Fig. 3:**
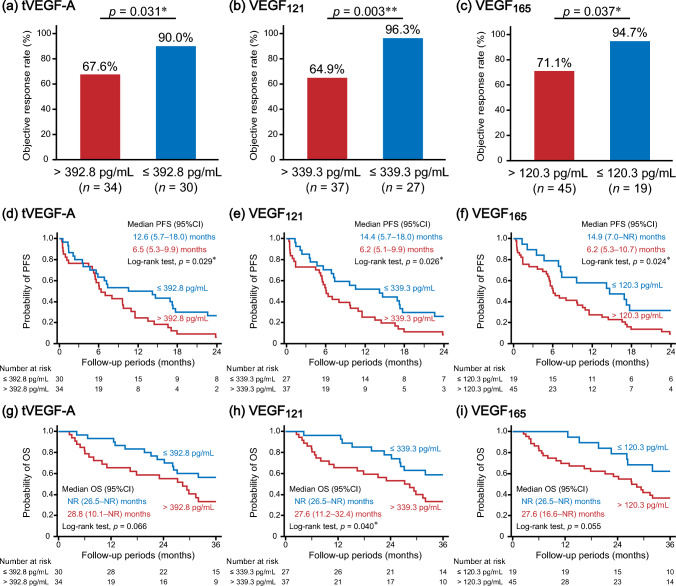
Comparison of objective response rate (ORR) and Kaplan–Meier analyses for progression-free survival (PFS) and overall survival (OS) according to serum levels of total vascular endothelial growth factor (tVEGF)-A and VEGF-A isoforms. The ORR was significantly lower in patients with higher serum levels of **a** tVEGF-A, **b** VEGF_121_, and **c** VEGF_165_ than in those with lower levels (*p* = 0.031, *p* = 0.003, and *p* = 0.037, respectively). Kaplan–Meier analysis showed that patients with higher serum levels of **d** tVEGF-A, **e** VEGF_121_, and **f** VEGF_165_ had a significantly shorter PFS than those with lower levels (*p* = 0.029, *p* = 0.026, and *p* = 0.024, respectively). Similarly, patients with higher serum levels of **h** VEGF_121_ had a significantly shorter OS than those with lower levels (*p* = 0.040), whereas **g** tVEGF-A and **i** VEGF_165_ showed similar trends without statistical significance (*p* = 0.066 and *p* = 0.055, respectively). ^*^p < 0.05 and ^**^p < 0.01, Pearson’s chi-square test or log-rank test. *Abbreviations*: NR, not reached; ORR, objective response rate; OS, overall survival; PFS, progression-free survival; tVEGF, total vascular endothelial growth factor; VEGF, vascular endothelial growth factor

When patients were stratified by these cut-off levels, significantly lower ORR and shorter PFS were observed in patients with higher levels of serum tVEGF-A, VEGF_121_, and VEGF_165_ than in those without (Fig. 3). Furthermore, only VEGF_121_ could stratify OS; Kaplan–Meier analysis showed a significantly shorter OS in patients with higher serum VEGF_121_ levels (> 339.3 pg/mL) than in those without (median OS 27.6 [95% CI: 11.2–32.4] months vs. not reached (NR) [95% CI: 26.5 months–NR], *p* = 0.040, respectively) (Fig. 3).

### Identification of independent factors related to ORR, PFS, and OS

Univariate logistic regression analysis revealed that among the serum tVEGF-A and VEGF-A isoform levels, only higher serum VEGF_121_ levels were significantly associated with a lower ORR. Furthermore, multivariate logistic regression analysis revealed that serum VEGF_121_ levels were independently associated with a lower ORR (Table 2).

**Table 2:** Identification of factors associated with objective response rate (ORR), progression-free survival (PFS), and overall survival (OS)

	ORR (Logistic regression analysis)			PFS (Cox hazard analysis)			OS (Cox hazard analysis)		
	OR	95% CI	*p*-value	HR	95% CI	*p*-value	HR	95% CI	*p*-value
**Univariate analysis**									
Age, years	1.014	0.964–1.064	0.586	0.993	0.971–1.017	0.569	1.005	0.976–1.037	0.769
Sex, male	0.889	0.268–3.065	0.848	0.902	0.521–1.559	0.711	0.926	0.460–1.866	0.830
Smoking history, current or former	0.889	0.268–3.065	0.848	0.845	0.487–1.467	0.550	0.903	0.448–1.820	0.775
BMI	0.996	0.836–1.198	0.969	0.999	0.920–1.080	0.982	1.031	0.931–1.135	0.540
ECOG PS, ≥ 2	0.104	0.013–0.607	0.012^*^	2.330	0.980–5.542	0.056	4.716	1.906–11.667	< 0.001^***^
Stage, III or recurrence	NA^†^	NA^†^	0.316	0.469	0.065–3.406	0.455	0.781	0.107–5.724	0.808
Histological type, adeno	NA^†^	NA^†^	0.480	0.866	0.118–6.326	0.887	NA^††^	NA^††^	0.999
* EGFR* mutation									
Exon 19 deletion	ref			ref			ref		
L858R mutation	0.333	0.079–1.222	0.098	0.997	0.560–1.777	0.993	1.293	0.607–2.753	0.506
Uncommon mutation	0.370	0.054–3.177	0.335	1.320	0.542–3.215	0.541	3.721	1.405–9.851	0.008^**^
Baseline tumor metastasis									
Brain metastasis, +	1.103	0.329–4.045	0.876	1.490	0.858–2.586	0.157	0.941	0.468–1.893	0.866
Pleural effusion, +	0.370	0.101–1.233	0.106	1.323	0.769–2.276	0.312	2.579	1.278–5.201	0.008^**^
Liver metastasis, +	0.805	0.199–4.080	0.774	1.146	0.588–2.234	0.690	2.008	0.926–4.352	0.077
EGFR-TKI generation									
First-generation	0.889	0.208–3.390	0.865	1.573	0.859–2.882	0.143	0.918	0.430–1.962	0.826
Second-generation	0.296	0.044–1.978	0.202	1.686	0.658–4.323	0.277	1.485	0.515–4.279	0.464
Third-generation	ref			ref			ref		
Serum tVEGF-A, per 100 pg/mL	0.915	0.800–1.021	0.110	1.035	0.984–1.078	0.132	1.053	0.986–1.108	0.079
Serum VEGF_121_, per 100 pg/mL	0.818	0.661–0.962	0.015^*^	1.110	1.027–1.187	0.004^**^	1.052	0.963–1.126	0.199
Serum VEGF_165_, per 100 pg/mL	0.762	0.505–1.036	0.083	1.090	0.948–1.216	0.172	1.155	0.953–1.335	0.089
**Multivariate analysis**									
Age, years	1.009	0.938–1.085	0.811	1.000	0.974–1.028	0.977	1.028	0.992–1.068	0.147
Sex, male	1.465	0.320–6.713	0.623	0.702	0.371–1.327	0.276	0.741	0.340–1.611	0.449
ECOG PS, ≥ 2	0.056	0.004–0.722	0.027^*^	1.323	0.483–3.621	0.586	6.604	2.013–21.661	0.002^**^
* EGFR* mutation									
Exon 19 deletion	ref			ref			ref		
L858R mutation	0.130	0.019–0.864	0.035^*^	1.013	0.519–1.978	0.970	1.648	0.666–4.075	0.280
Uncommon mutation	0.315	0.031–3.247	0.332	1.838	0.703–4.809	0.215	6.068	1.953–18.849	0.002^**^
Baseline tumor metastasis									
Brain metastasis, +	2.209	0.383–12.731	0.489	1.080	0.564–2.069	0.817	0.710	0.278–1.814	0.475
EGFR-TKI generation									
First-generation	1.081	0.196–5.956	0.929	2.070	1.060–4.045	0.033^*^	1.007	0.418–2.427	0.988
Second-generation	0.317	0.021–4.866	0.410	2.237	0.781–6.406	0.134	2.421	0.686–8.541	0.169
Third-generation	ref			ref			ref		
Serum VEGF_121_, per 100 pg/mL	0.786	0.582–0.972	0.025^*^	1.149	1.050–1.248	0.001^**^	1.090	0.979–1.200	0.092

^*^p < 0.05, ^**^p < 0.01, and ^***^p < 0.001, logistic regression analysis or Cox proportional hazard model

^†^OR and 95% CI could not be calculated because only one group responded to EGFR-TKI

^††^HR and 95% CI could not be calculated because no event occurred in one group

Adeno, adenocarcinoma; BMI, body mass index; CI, confidence interval; ECOG PS, Eastern Cooperative Oncology Group Performance Status; EGFR, epidermal growth factor receptor; HR, hazard ratio; NA, not available; OR, odds ratio; OS, overall survival; TKI, tyrosine kinase inhibitor; tVEGF, total vascular endothelial growth factor; VEGF, vascular endothelial growth factor

Moreover, univariate and multivariate Cox proportional hazards models revealed that higher serum VEGF_121_ levels were independently associated with shorter PFS (Table 2). In contrast, no significant association was observed between serum VEGF_121_ levels and OS (Table 2).

To evaluate the impact of potential confounders of VEGF, we additionally performed logistic and Cox regression analyses including WBC count, platelet count, D-dimer, and CRP as explanatory variables (Supplementary Table 2). In consideration of potential multicollinearity because of correlations and biological interrelatedness between these markers and VEGF, each variable was included in the multivariate analyses instead of VEGF. In the multivariable models, only D-dimer was significantly associated with ORR, although it was not significant in the univariate models.

### Subgroup analyses based on EGFR-TKI generation

In the subgroup analyses, the cohort was divided into two groups: patients treated with first-generation EGFR-TKIs and those treated with second- or third-generation EGFR-TKIs. The associations of ORR and 2-year PFS rate with serum levels of tVEGF-A, VEGF_121_, and VEGF_165_ were compared. In both treatment groups, patients with higher serum tVEGF-A, VEGF_121_, and VEGF_165_ levels consistently showed lower ORR and 2-year PFS rate than those with lower levels (Table 3). Notably, although similar trends were observed in patients treated with first-generation EGFR-TKIs, the differences were more pronounced in those who received second- or third-generation EGFR-TKIs.

**Table 3:** Comparison of objective response rate (ORR) and 2-year progression-free survival (PFS) rate according to serum total vascular endothelial growth factor (tVEGF)-A and VEGF-A isoforms levels

	Second- or third-generation EGFR-TKI (*n* = 29)		First-generation EGFR-TKI (*n* = 35)	
	ORR	2-year PFS rate	ORR	2-year PFS rate
Serum tVEGF-A				
High (> 392.8 pg/mL)	62.5%	6.3%	72.2%	6.0%
Low (≤ 392.8 pg/mL)	92.3%	46.2%	88.2%	11.8%
Serum VEGF_121_				
High (> 339.3 pg/mL)	63.2%	10.5%	66.7%	6.1%
Low (≤ 339.3 pg/mL)	100.0%	50.0%	94.1%	11.8%
Serum VEGF_165_				
High (> 120.3 pg/mL)	69.6%	13.0%	72.7%	4.9%
Low (≤ 120.3 pg/mL)	100.0%	66.7%	92.3%	15.4%

EGFR, epidermal growth factor receptor; ORR, objective response rate; PFS, progression-free survival; TKI, tyrosine kinase inhibitor; tVEGF, total vascular endothelial growth factor; VEGF, vascular endothelial growth factor

## Discussion

In this study, we evaluated whether serum tVEGF-A and VEGF-A isoforms, VEGF_121_ and VEGF_165_, could be associated with the treatment efficacy and prognosis in patients with previously untreated *EGFR*-mutated advanced NSCLC treated with EGFR-TKI monotherapy. Only serum VEGF_121_ levels were significantly higher in non-responders than in responders. Additionally, although tVEGF-A, VEGF_121_, and VEGF_165_ could stratify ORR and PFS as shown in Fig. 3, only VEGF_121_ was able to stratify OS. Furthermore, multivariate analyses showed that only higher serum VEGF_121_ levels were independently associated with lower ORR and shorter PFS. These findings suggest that serum VEGF_121_ may serve as a potential biomarker associated with the efficacy of EGFR-TKI and prognosis in patients with *EGFR*-mutated NSCLC treated with EGFR-TKIs.

Several biological features support the present data of VEGF in the efficacy of EGFR-TKIs in patients with *EGFR*-mutated NSCLC. First, the EGFR and VEGF pathways share overlapping downstream cascades (e.g., PI3K/AKT and RAS/RAF/ERK), and also operate independently during tumor progression [4, 17]. EGFR signaling enhances VEGF-A transcription via two mechanisms: activation of the PI3K/AKT pathway and hypoxia-inducible factor-1α-mediated transcriptional upregulation [18]. In line with this, VEGF expression has been reported to be upregulated in *EGFR*-mutated NSCLC cells and tissues [3]. VEGF is a key pro-angiogenic mediator that promotes tumor growth and metastasis [19], and therefore, VEGF upregulation has been implicated in resistance to EGFR-TKIs [20]. Second, VEGF increases vascular permeability and elevates interstitial fluid pressure, thereby impairing intratumoral drug delivery [21–23]. Agents targeting the VEGF/VEGFR pathway can normalize abnormal tumor vessels by diminishing hyperpermeability, increasing pericyte coverage, and restoring the basement membrane [21, 23]. In lung cancer models, adding an anti-angiogenic agent to erlotinib normalized tumor vasculature and increased intratumoral erlotinib concentrations compared with erlotinib alone [24]. Collectively, these data provide a coherent rationale that elevated circulating VEGF may reflect the activation of the EGFR-VEGF pathway crosstalk and poor drug penetration, and therefore, higher levels of serum VEGF-A and its isoforms are associated with lower EGFR-TKI efficacy, as shown in Fig. 3.

This study also showed that only higher serum VEGF_121_ levels were independently associated with a lower ORR and shorter PFS, whereas tVEGF-A and VEGF_165_ were not (Table 2). VEGF_121_ promotes tumor angiogenesis and increases peritumoral vascular permeability [14, 25–27], and has been shown to induce lymphangiogenesis in sentinel lymph nodes [28]. Additionally, across multiple cancers, high tumor VEGF_121_ expression has been associated with poor prognosis [29–31]. These findings support the notion that VEGF_121_, which is a more specific isoform involved in tumor growth, is a potential biomarker; however, external validation is needed.

A previous study examining the association between pre-treatment serum VEGF levels and the efficacy of EGFR-TKIs has included only first-generation EGFR-TKIs (gefitinib or icotinib) and found no significant relationship between VEGF levels and PFS [8]. In contrast, the present study included patients treated with second- and third-generation EGFR-TKIs, in addition to those treated with first-generation EGFR-TKIs. Therefore, we stratified the patients into first-generation and second- or third-generation EGFR-TKI groups and compared the treatment efficacy according to the baseline serum tVEGF-A and VEGF-A isoform levels. In both treatment groups, patients with higher baseline VEGF-A or VEGF-A isoform levels exhibited poorer ORR and shorter PFS than those with lower levels, but this difference was more pronounced in patients treated with second- or third-generation EGFR-TKIs. These data may explain why our study, including patients treated with second- or third-generation EGFR-TKIs, was able to identify a clear difference in the efficacy of EGFR-TKIs.

In treatment-naïve patients, the efficacy of combining EGFR-TKIs with VEGF/VEGFR inhibitors varies depending on the generation of EGFR-TKIs. For first-generation EGFR-TKIs, the addition of VEGF/VEGFR inhibitors has demonstrated consistent benefits, as shown in the NEJ026 trial (erlotinib plus bevacizumab) and the RELAY trial (erlotinib plus ramucirumab) [5, 6]. In contrast, second-generation EGFR-TKIs have shown no significant additive benefit from VEGF inhibition, as seen in the AfaBev-CS study (afatinib plus bevacizumab) [32]. For third-generation EGFR-TKIs, the addition of VEGF/VEGFR inhibitors has produced controversial results; the WJOG9717L trial (osimertinib plus bevacizumab) showed no significant additional benefit, whereas the RAMOSE trial (osimertinib plus ramucirumab) demonstrated a potential advantage of combination therapy [33, 34]. Collectively, these results suggest that the additive benefit of VEGF/VEGFR inhibition appears more reproducible with first-generation TKIs, whereas for second- and third-generation TKIs, the magnitude of benefit may depend on patient population characteristics, and therefore, patients who should be treated with co-administration of VEGF/VEGFR inhibitors need to be selected [35]. In the exploratory biomarker analyses of the phase II JO25567 study, erlotinib plus bevacizumab showed favorable treatment outcomes in patients with low plasma VEGF-A levels [36]. Furthermore, in a previous study of patients with metastatic colorectal cancer, low plasma VEGF_121_ levels were identified as a potential biomarker to predict which patients would benefit from the addition of bevacizumab [37]. From this perspective, serum VEGF_121_ may warrant further evaluation as a stratification marker in future studies of EGFR-TKI plus VEGF/VEGFR inhibitor therapy.

The association between higher levels of serum VEGF_121_ and poor treatment outcome may not be specific for *EGFR*-mutated NSCLC. First, serum tVEGF-A and its isoforms were significantly correlated with WBC count, platelet count, and CRP. These findings raise the possibility that circulating VEGF-A may reflect systemic inflammation rather than tumor-specific biology in *EGFR*-mutated NSCLC. Second, our previous study has shown that the higher levels of serum VEGF_121_ are associated with shorter PFS in patients with NSCLC receiving ICI [15]. These data suggest VEGF_121_ may have potential as a prognostic factor in patients with NSCLC, but not a predictive biomarker specific for the efficacy of EGFR-TKI, although further investigation is warranted by focusing on each type of anti-neoplastic agent.

This study had several limitations that should be considered. First, this was a retrospective study with a limited sample size. Second, we evaluated only patients treated with EGFR-TKI monotherapy in this study. Given that combination therapies of EGFR-TKI with VEGF/VEGFR inhibitors, chemotherapy, and bispecific antibody are now approved, the utility of serum VEGF_121_ should be validated in cohorts receiving these regimens. Third, although third-generation EGFR-TKIs are currently the standard treatment for *EGFR*-mutated NSCLC, this study included only a limited number of patients who received these agents. Serum VEGF-A and its isoforms may have potential utility for stratifying ORR and PFS in third-generation EGFR-TKI treatment, which warrants further investigation in larger cohorts. Fourth, although VEGF_121_ showed the highest AUC among the evaluated biomarkers, its discriminatory performance (AUC 0.678) and relatively low sensitivity (52.0%) suggest that its clinical utility remains limited. In addition, the biological roles of VEGF_121_ in *EGFR*-mutated NSCLC remain incompletely understood. Further studies are warranted to elucidate the underlying mechanisms and their prognostic relevance.

In conclusion, serum VEGF_121_ may have potential as a prognostic biomarker in patients with *EGFR*-mutated NSCLC treated with EGFR-TKIs, although a future validation study is necessary.

## Data Availability

The data supporting the findings of this study are available from the corresponding author upon reasonable requests.

## Abbreviation

Adeno: Adenocarcinoma
Adenosquamous: Adenosquamous carcinoma
AUC: Area under the curve
BMI: Body mass index
CI: Confidence interval
CR: Complete response
CRP: C-reactive protein
ECOG PS: Eastern Cooperative Oncology Group Performance Status
EGFR: Epidermal growth factor receptor
ELISA: Enzyme-linked immunosorbent assay
HR: Hazard ratio
ICI: Immune checkpoint inhibitor
IQR: Interquartile range
NA: Not available
NE: Not evaluable
NR: Not reached
NSCLC: Non-small cell lung cancer
OR: Odds ratio
ORR: Objective response rate
OS: Overall survival
PBS: Phosphate-buffered saline
PD: Progressive disease
PFS: Progression-free survival
PR: Partial response
ROC: Receiver operating characteristic
SD: Stable disease
TKI: Tyrosine kinase inhibitor
tVEGF: Total vascular endothelial growth factor
VEGF: Vascular endothelial growth factor
VEGFR: Vascular endothelial growth factor receptor
WBC: White blood cell

## Declarations

### Conference presentation

Portions of this work were presented at the 66th Annual Meeting of the Japan Lung Cancer Society (Oral Session, November 8, 2025).
