# Correction: Clinical implications of serum total VEGF-A and VEGF-A isoforms in patients with EGFR-mutated advanced non-small cell lung cancer treated with EGFR-TKIs

**DOI:** 10.1007/s10147-026-03106-y

**Published:** 2026-07-06

**Authors:** Tetsu Hirakawa, Kakuhiro Yamaguchi, Kiyofumi Shimoji, Shinjiro Sakamoto, Yasushi Horimasu, Takeshi Masuda, Taku Nakashima, Hiroshi Iwamoto, Hironobu Hamada, Noboru Hattori

**Affiliations:** 1 Department of Molecular and Internal Medicine, Graduate School of Biomedical and Health Sciences, Hiroshima University, 1-2-3 Kasumi, Minami-Ku, Hiroshima, 734-8551 Japan; 2 Department of Respiratory Medicine, Hiroshima University Hospital, Hiroshima, Japan; 3 Department of Physical Analysis and Therapeutic Sciences, Graduate School of Biomedical and Health Sciences, Hiroshima University, Hiroshima, Japan

**Correction to: International Journal of Clinical Oncology**

10.1007/s10147-026-03083-2

In Table 2 of this article, the values under the column header ‘PFS (Cox hazard analysis)’ were inadvertently omitted. For completeness and transparency, the old incorrect version and the corrected version of Table 2 are displayed below.

Incorrect Table 2:

**Table 2:** Identification of factors associated with objective response rate (ORR), progression-free survival (PFS), and overall survival (OS)

	ORR Logistic regression analysis)			OS (Cox hazard analysis)		
	OR	95% CI	*p*-value	HR	95% CI	*p*-value
Univariate analysis						
Age, years	1.014	0.964–1.064	0.586	1.005	0.976–1.037	0.769
Sex, male	0.889	0.268–3.065	0.848	0.926	0.460–1.866	0.830
Smoking history, current or former	0.889	0.268–3.065	0.848	0.903	0.448–1.820	0.775
BMI	0.996	0.836–1.198	0.969	1.031	0.931–1.135	0.540
ECOG PS, ≥ 2	0.104	0.013–0.607	0.012^*^	4.716	1.906–11.667	<0.001^***^
Stage, III or recurrence	NA^†^	NA^†^	0.316	0.781	0.107–5.724	0.808
Histological type, adeno	NA^†^	NA^†^	0.480	NA^††^	NA^†^	0.999
*EGFR *mutation						
Exon 19 deletion	ref	ref				
L858R mutation	0.333	0.079–1.222	0.098	1.293	0.607–2.753	0.506
Uncommon mutation	0.370	0.054–3.177	0.335	3.721	1.405–9.851	0.008^**^
Baseline tumor metastasis						
Brain metastasis, +	1.103	0.329–4.045	0.876	0.941	0.468–1.893	0.866
Pleural effusion, +	0.370	0.101–1.233	0.106	2.579	1.278–5.201	0.008^**^
Liver metastasis, +	0.805	0.199–4.080	0.774	2.008	0.926–4.352	0.077
EGFR-TKI generation						
First-generation	0.889	0.208–3.390	0.865	0.918	0.430–1.962	0.826
Second-generation	0.296	0.044–1.978	0.202	1.485	0.515–4.279	0.464
Third-generation	ref	ref				
Serum tVEGF-A, per 100 pg/mL	0.915	0.800–1.021	0.110	1.053	0.986–1.108	0.079
Serum VEGF_121_, per 100 pg/mL	0.818	0.661–0.962	0.015^*^	1.052	0.963–1.126	0.199
Serum VEGF_165_, per 100 pg/mL	0.762	0.505–1.036	0.083	1.155	0.953–1.335	0.089
Multivariate analysis						
Age, years	1.009	0.938–1.085	0.811	1.028	0.992–1.068	0.147
Sex, male	1.465	0.320–6.713	0.623	0.741	0.340–1.611	0.449
ECOG PS, ≥ 2	0.056	0.004–0.722	0.027^*^	6.604	2.013–21.661	0.002^**^
*EGFR *mutation						
Exon 19 deletion	ref	ref				
L858R mutation	0.130	0.019–0.864	0.035^*^	1.648	0.666–4.075	0.280
Uncommon mutation	0.315	0.031–3.247	0.332	6.068	1.953–18.849	0.002^**^
Baseline tumor metastasis						
Brain metastasis, +	2.209	0.383–12.731	0.489	0.710	0.278–1.814	0.475
EGFR-TKI generation						
First-generation	1.081	0.196–5.956	0.929	1.007	0.418–2.427	0.988
Second-generation	0.317	0.021–4.866	0.410	2.421	0.686–8.541	0.169
Third-generation	ref	ref				
Serum VEGF_121_, per 100 pg/mL	0.786	0.582–0.972	0.025^*^	1.090	0.979–1.200	0.092

^*^p < 0.05, ^**^p < 0.01, and ^***^p < 0.001, logistic regression analysis or Cox proportional hazard model

^†^OR and 95% CI could not be calculated because only one group responded to EGFR-TKI

^††^HR and 95% CI could not be calculated because no event occurred in one group

Correct Table 2:

**Table 2:** Identification of factors associated with objective response rate (ORR), progression-free survival (PFS), and overall survival (OS)

	ORR (Logistic regression analysis)			PFS (Cox hazard analysis)			OS (Cox hazard analysis)		
	OR	95% CI	*p*-value	HR	95% CI	*p*-value	HR	95% CI	*p*-value
Univariate analysis									
Age, years	1.014	0.964–1.064	0.586	0.993	0.971–1.017	0.569	1.005	0.976–1.037	0.769
Sex, male	0.889	0.268–3.065	0.848	0.902	0.521–1.559	0.711	0.926	0.460–1.866	0.830
Smoking history, current or former	0.889	0.268–3.065	0.848	0.845	0.487–1.467	0.550	0.903	0.448–1.820	0.775
BMI	0.996	0.836–1.198	0.969	0.999	0.920–1.080	0.982	1.031	0.931–1.135	0.540
ECOG PS, ≥ 2	0.104	0.013–0.607	0.012^*^	2.330	0.980–5.542	0.056	4.716	1.906–11.667	< 0.001^***^
Stage, III or recurrence	NA^†^	NA^†^	0.316	0.469	0.065–3.406	0.455	0.781	0.107–5.724	0.808
Histological type, adeno	NA^†^	NA^†^	0.480	0.866	0.118–6.326	0.887	NA^††^	NA^††^	0.999
*EGFR* mutation									
Exon 19 deletion	ref			ref			ref		
L858R mutation	0.333	0.079–1.222	0.098	0.997	0.560–1.777	0.993	1.293	0.607–2.753	0.506
Uncommon mutation	0.370	0.054–3.177	0.335	1.320	0.542–3.215	0.541	3.721	1.405–9.851	0.008^**^
Baseline tumor metastasis									
Brain metastasis, +	1.103	0.329–4.045	0.876	1.490	0.858–2.586	0.157	0.941	0.468–1.893	0.866
Pleural effusion, +	0.370	0.101–1.233	0.106	1.323	0.769–2.276	0.312	2.579	1.278–5.201	0.008^**^
Liver metastasis, +	0.805	0.199–4.080	0.774	1.146	0.588–2.234	0.690	2.008	0.926–4.352	0.077
EGFR-TKI generation									
First-generation	0.889	0.208–3.390	0.865	1.573	0.859–2.882	0.143	0.918	0.430–1.962	0.826
Second-generation	0.296	0.044–1.978	0.202	1.686	0.658–4.323	0.277	1.485	0.515–4.279	0.464
Third-generation	ref			ref			ref		
Serum tVEGF-A, per 100 pg/mL	0.915	0.800–1.021	0.110	1.035	0.984–1.078	0.132	1.053	0.986–1.108	0.079
Serum VEGF_121_, per 100 pg/mL	0.818	0.661–0.962	0.015^*^	1.110	1.027–1.187	0.004^**^	1.052	0.963–1.126	0.199
Serum VEGF_165_, per 100 pg/mL	0.762	0.505–1.036	0.083	1.090	0.948–1.216	0.172	1.155	0.953–1.335	0.089
Multivariate analysis									
Age, years	1.009	0.938–1.085	0.811	1.000	0.974–1.028	0.977	1.028	0.992–1.068	0.147
Sex, male	1.465	0.320–6.713	0.623	0.702	0.371–1.327	0.276	0.741	0.340–1.611	0.449
ECOG PS, ≥ 2	0.056	0.004–0.722	0.027^*^	1.323	0.483–3.621	0.586	6.604	2.013–21.661	0.002^**^
*EGFR* mutation									
Exon 19 deletion	ref			ref			ref		
L858R mutation	0.130	0.019–0.864	0.035^*^	1.013	0.519–1.978	0.970	1.648	0.666–4.075	0.280
Uncommon mutation	0.315	0.031–3.247	0.332	1.838	0.703–4.809	0.215	6.068	1.953–18.849	0.002^**^
Baseline tumor metastasis									
Brain metastasis, +	2.209	0.383–12.731	0.489	1.080	0.564–2.069	0.817	0.710	0.278–1.814	0.475
EGFR-TKI generation									
First-generation	1.081	0.196–5.956	0.929	2.070	1.060–4.045	0.033^*^	1.007	0.418–2.427	0.988
Second-generation	0.317	0.021–4.866	0.410	2.237	0.781–6.406	0.134	2.421	0.686–8.541	0.169
Third-generation	ref			ref			ref		
Serum VEGF_121_, per 100 pg/mL	0.786	0.582–0.972	0.025^*^	1.149	1.050–1.248	0.001^**^	1.090	0.979–1.200	0.092

^*^p < 0.05, ^**^p < 0.01, and ^***^p < 0.001, logistic regression analysis or Cox proportional hazard model

^†^OR and 95% CI could not be calculated because only one group responded to EGFR-TKI

^††^HR and 95% CI could not be calculated because no event occurred in one group

The original article has been corrected.

